# RNA Tertiary Interactions in a Riboswitch Stabilize the Structure of a Kink Turn

**DOI:** 10.1016/j.str.2011.07.003

**Published:** 2011-09-07

**Authors:** Kersten T. Schroeder, Peter Daldrop, David M.J. Lilley

**Affiliations:** 1 Cancer Research UK Nucleic Acid Structure Research Group, MSI/WTB Complex, The University of Dundee, Dow Street, Dundee DD1 5EH, UK

## Abstract

The kink turn is a widespread RNA motif that introduces an acute kink into the axis of duplex RNA, typically comprising a bulge followed by a G⋅A and A⋅G pairs. The kinked conformation is stabilized by metal ions, or the binding of proteins including L7Ae. We now demonstrate a third mechanism for the stabilization of k-turn structure, involving tertiary interactions within a larger RNA structure. The SAM-I riboswitch contains an essential standard k-turn sequence that kinks a helix so that its terminal loop can make a long-range interaction. We find that some sequence variations in the k-turn within the riboswitch do not prevent SAM binding, despite preventing the folding of the k-turn in isolation. Furthermore, two crystal structures show that the sequence-variant k-turns are conventionally folded within the riboswitch. This study shows that the folded structure of the k-turn can be stabilized by tertiary interactions within a larger RNA structure.

## Graphical abstract

**Graphical Abstract:**

**Figure:**
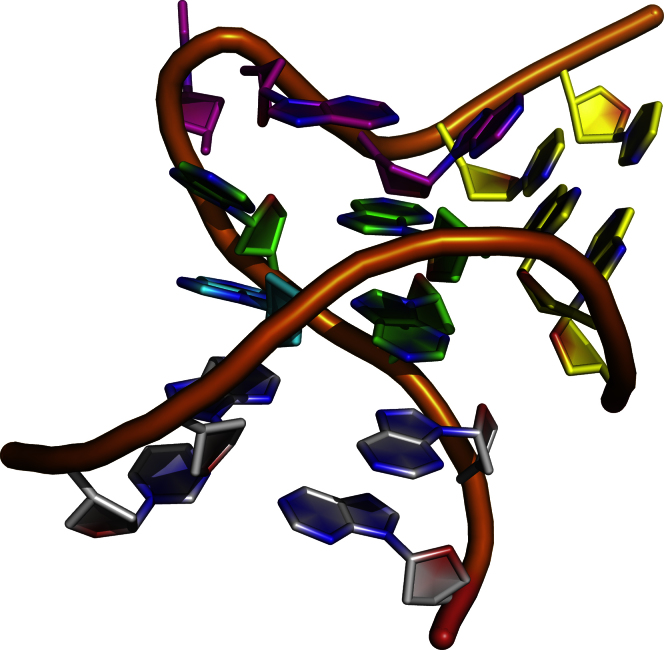

**Highlights:**

► The folded conformation of a kink turn is stabilized by long-range tertiary interactions in RNA ► The SAM-I riboswitch contains a k-turn, that folds in the presence of Mg^2+^ ions in isolation ► Nucleotide substitution in the G•A pairs of the k-turn prevent folding in isolation, but some can be stabilized in the context of the riboswitch, allowing binding of SAM ligand ► The crystal structure of a SAM-I riboswitch with a variant k-turn provides the first example of a k-turn with an A•A pair at the 2b•2n position

## Introduction

**Figure 1:**
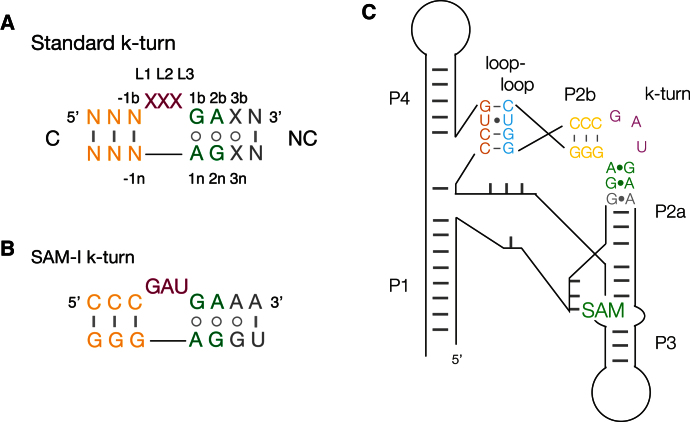
The SAM-I Riboswitch and Its k-Turn (A) The sequence of a standard k-turn, and the nomenclature for nucleotide positions (Liu and Lilley, 2007). (B) The sequence of the k-turn found in the SAM-I riboswitch of *T. tengcongensis*. (C) A schematic drawing of the secondary structure of the *T. tengcongensis* SAM-I riboswitch (Montange and Batey, 2006), showing the position of the k-turn, the loop-loop interaction and the location of SAM binding. The gray bars merely indicate base-paired regions, and the number of these do not accurately represent the number of base pairs.

The kink-turn (k-turn) is a very common structural motif in RNA that contributes to long-range architecture of RNA by virtue of introducing an acute kink into the RNA helix. Both subunits of the ribosome contain multiple k-turns (Klein et al., 2001), and k-turns are also found in snoRNA species of box C/D and H/ACA classes (Hamma and Ferré-D'Amaré, 2004; Moore et al., 2004), U4 snRNA (Vidovic et al., 2000; Woźniak et al., 2005), and untranslated regions of mRNA including riboswitches (Blouin and Lafontaine, 2007; Heppell and Lafontaine, 2008; Montange and Batey, 2006). k-turns are therefore involved in virtually all aspects of RNA function, including translation, guided methylation and pseudouridylation of RNA, spliceosome assembly, and genetic control. A comprehensive database of sequence and structural information on k-turns is available at http://www.dundee.ac.uk/biocentre/nasg/kturn/ (Schroeder et al., 2010).

k-turns introduce a discontinuity in double helical RNA. The standard k-turn comprises a three nucleotide bulge flanked on its 3′ side by G⋅A, A⋅G and possibly further non-Watson-Crick base pairs (the noncanonical or NC helix), and on its 5′ side by regular base pairing (the C helix) (Figures 1A and 1B). The G⋅A pairs are *trans* sugar edge-Hoogsteen edge base pairs that direct the minor groove edges of the two adenine bases toward the minor groove of the C helix. Intimate A-minor interactions and a number of specific hydrogen bonds (Liu and Lilley, 2007; Turner and Lilley, 2008) stabilize a kink with an included angle of close to 60°.

Perhaps surprisingly, an isolated k-turn RNA does not adopt the tightly kinked geometry in the absence of added metal ions (Goody et al., 2004; Matsumura et al., 2003), but rather forms a more extended structure typical of a normal three nucleotide bulge (Gohlke et al., 1994). Addition of Mg^2+^ ions induces folding into the structure observed in the crystal in a noncooperative two-state process with a midpoint of the order of 100 μM (Liu and Lilley, 2007). Folding is also induced by significantly higher concentrations of monovalent ions, suggesting that specific ion binding is not required; instead, phosphate charge neutralization by counter ions results in folding into the kinked structure.

There is a second way in which the folding of the k-turn can be induced. k-turns frequently serve as binding sites for proteins, and binding can result in folding even in the absence of added metal ions. Archaeal L7Ae and related proteins (such as human 15.5 kDa protein (Nottrott et al., 1999)) have been particularly well studied in this regard (Rozhdestvensky et al., 2003; Szewczak et al., 2002; Vidovic et al., 2000). We have shown that L7Ae induces folding of several k-turns (Turner et al., 2005), binding with an extremely high apparent affinity of the order of 10 pM (Turner and Lilley, 2008). This is consistent with one role (of many) for this family of proteins in assembly of the box C/D complex, which is initiated by the binding of the L7Ae-related protein to a k-turn (Ganot et al., 1997; Kiss-László et al., 1996). The human 15.5 kDa protein binds to the k-turn of U4 snRNA (Vidovic et al., 2000; Woźniak et al., 2005).

In principle, a third process might lead to stabilization of the kinked conformation of the k-turn. The sharp change in helical trajectory at the turn is frequently used to create long-range architecture in large RNA molecules. Conversely, tertiary interactions in such species might stabilize the kinked geometry of the k-turn, so that in a sense the RNA structure forces the k-turn to adopt its folded conformation. We therefore set out to investigate this possibility, and for this purpose we chose the SAM riboswitch as a model system.

Riboswitches are *cis*-acting elements occurring in mRNA of principally prokaryotic organisms, that bind small molecules in a manner that leads to metabolic autoregulation (Roth and Breaker, 2009). Most riboswitches (but not all) can exist in two mutually exclusive conformations, one of which lowers gene expression either by modulating transcriptional termination or initiation of translation. The balance between the stability of the two conformations is influenced by the binding of the small molecule that is somehow functionally related to the gene product. There are a number of riboswitches that bind S-adenosyl methionine (SAM) to regulate methionine biosynthesis (Winkler et al., 2003), falling into five structural classes. The SAM-I riboswitch is based around an elaborated four way helical junction (Figure 1C); the crystal structure of the SAM-I riboswitch of *Thermoanaerobacter tengcongensis* was solved by Batey and colleagues (Montange and Batey, 2006), showing that the junction creates a pocket for the binding of SAM. Two adjacent helical arms of the junction make a tertiary contact in which the terminal loop of one helix (P2) base pairs with an internal loop within the other (P4). In order to do this, helix P2 must be severely kinked, and this is facilitated by a k-turn. This sequence is a good match to the k-turn consensus; it is strongly conserved and its disruption was shown to lower SAM binding affinity (Heppell and Lafontaine, 2008).

We now turn this around to ask if the tertiary contacts formed in the SAM-I riboswitch can stabilize a k-turn with a less than optimal sequence such that it cannot fold in isolated RNA by the addition of Mg^2+^ ions alone. In the first instance, we test the folding of the complete riboswitch indirectly by examining its ability to bind SAM, measured by calorimetry. We then use X-ray crystallography to show that such impaired k-turns can be correctly folded into a conventional k-turn structure. We conclude that tertiary interaction does provide another mechanism for stabilizing the folded k-turn conformation.

## Results

### The SAM Riboswitch k-Turn

The k-turn of the *T. tengcongensis* SAM-I riboswitch has a very standard sequence, close to the consensus (Figure 1B). It has a three base bulge followed by G⋅A and A⋅G pairs at the 1b⋅1n and 2b⋅2n positions, with an additional A⋅G pair at the 3b⋅3n position. It is closely similar to the near-consensus Kt-7 sequence. The backbone atoms (O5′, C5′, C4′, C3′, O3′, P and the nonbridging phosphate O) of the riboswitch k-turn structure (PDB file: 3gx5) (Montange et al., 2010) are superimposable with those of *Haloarcula marismortui* Kt-7 (PDB file: 3cc2) (Blaha et al., 2008), with an rmsd of 1.4 Å. All the key hydrogen-bonding interactions are present, including those of the G⋅A pairs, the L1 O2′ to A1n N1 and the L3 O2′ to the *pro*S nonbridging O of the phosphate linking L1 and L2. In all respects this is a very standard k-turn that should be induced to fold in isolation in the presence of Mg^2+^ ions. The k-turns of the SAM-I riboswitches of *Bacillus subtilis* (PDB file: 3npb) (Lu et al., 2010) and *T. tengcongensis* are also very similar; the backbone atoms align with an rmsd of 1.0 Å.

### The Isolated k-Turn Element Is Induced to Fold by Mg^2+^ Ions

**Figure 2:**
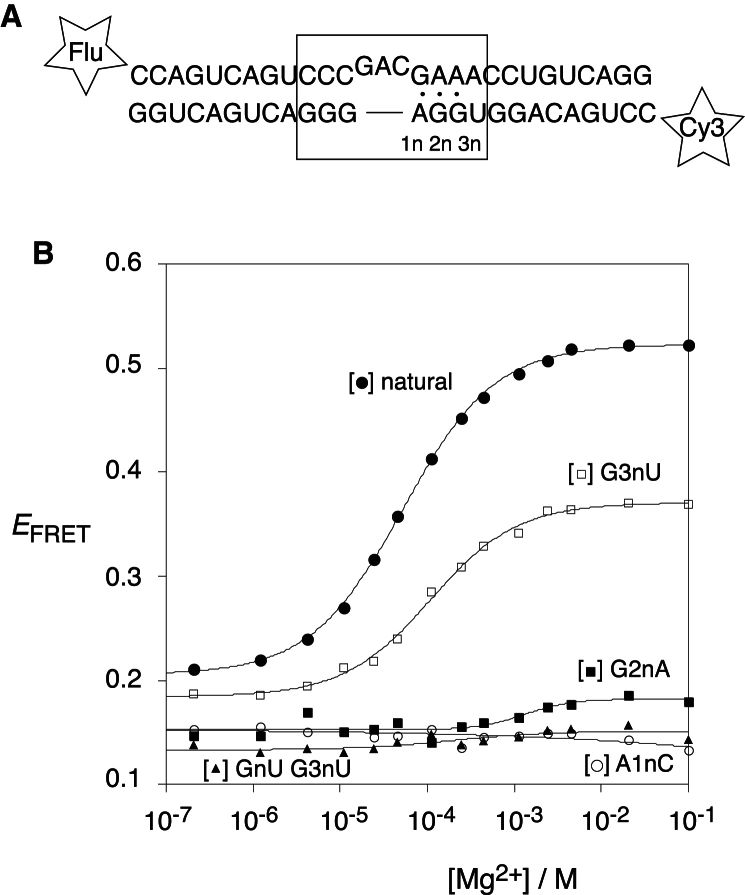
Folding of the SAM-I Riboswitch k-Turn as an Isolated RNA Duplex The k-turn sequence and variants were located centrally in a 25 bp duplex, terminally labeled with fluorescein (Flu) and Cy3 (Cy3) fluorophores. FRET efficiency (*E*_FRET_) was measured in the steady state as a function of Mg^2+^ ion concentration. (A) The sequence of the RNA duplex containing the natural sequence k-turn. Other substitutions were included as indicated. (B) Plot of *E*_FRET_ as a function of Mg^2+^ ion concentration. The data have been fitted to a model for a two-state transition (Equation (1)) (lines). Data: Filled circles, natural sequence k-turn; open circles, A1nC substitution; filled squares, G2nA substitution; open squares, G3nU substitution; filled triangles; G2nU, G3nU double substitution. Data for further variants are presented in Figure S1. See also Table S1.

We have studied the possible Mg^2+^-induced folding of the k-turn of the SAM-I riboswitch as an isolated species. For this purpose, we constructed an RNA duplex of 25 bp with a central sequence corresponding to the riboswitch k-turn. In order to study this by fluorescence resonance energy transfer (FRET), fluorescein (donor) and Cy3 (acceptor) fluorophores were attached to the 5′ termini (Figure 2A). Folding the k-turn into its kinked conformation brings the fluorophores closer together, increasing the efficiency of energy transfer from the fluorescein to Cy3.

The efficiency of FRET (*E*_FRET_) was measured by steady-state fluorimetry, using the acceptor normalization method (Clegg, 1992). *E*_FRET_ is plotted as a function of Mg^2+^ ion concentration for the SAM-I riboswitch k-turn in Figure 2B. The unmodified k-turn sequence clearly undergoes an ion-induced structural change, with an increase in FRET efficiency corresponding to a marked kinking of the helix as expected for the formation of the k-turn. The data have been fitted to a simple two-state binding model, giving a change in FRET efficiency Δ*E*_FRET_ = 0.32 ± 0.03 and a transition midpoint [Mg^2+^]1/2 = 50 ± 8 μM (see Table S1 available online). The Hill coefficient n = 0.8 ± 0.1. These values are similar to those of a standard k-turn such as Kt-7, and indicate that the folding into the tightly kinked structure occurs in response to the noncooperative binding of Mg^2+^ ions.

We also examined the behavior of variant k-turn sequences, containing sequence substitutions in the non-Watson-Crick pairs in the NC helix, multiply or singly. The triple substitution A1nC, G2nU, G3nU converts the k-turn into a simple three nucleotide bulge. Unsurprisingly, this variant exhibited no increase in *E*_FRET_ with addition of Mg^2+^ ions, i.e., no ion-induced folding into the k-turn conformation (Figure S1). We therefore selectively modified the non-Watson-Crick pairs either singly or pairwise. The single substitutions A1nC and G2nA prevented significant Mg^2+^ ion-induced folding (Figure 2B). This was also true for the substitutions A1nU and G2nC (Figure S1), as well as the double substitution G2nU, G3nU (Figure 2B). We also examined the importance of the third non-Watson-Crick pair at the 3b⋅3n position, making a G3nU variant that creates a potential A-U base pair at the third position in the NC helix. This resulted in partially impaired folding, with Δ*E*_FRET_ = 0.19 ± 0.02, [Mg^2+^]1/2 = 110 ± 12 μM and n = 0.9 ± 0.1. The lower amplitude of the transition indicates that the nature of the base pair at the 3b⋅3n position contributes to the stability of the folded k-turn, as we have previously found for *T. thermophilus* Kt-23 (Schroeder and Lilley, 2009), although the substitution at the 3n position is clearly less inhibitory to folding than changes to the first two positions of the NC helix.

In summary, metal ion-induced folding of the SAM-I k-turn in isolation requires the retention of the G⋅A pairs at the 1b⋅1n and 2b⋅2n positions, and is partially destabilized by alteration of the pair at the 3b⋅3n position.

### Binding of SAM to the Riboswitch Studied by Calorimetry

Having established that the k-turn of the SAM-I riboswitch is induced to fold in isolation by addition of Mg^2+^ ions, but that this is prevented by substitution in the 1b⋅1n and 2b⋅2n positions, we sought to study the structural function of the k-turn in situ in the riboswitch. We employed an indirect reporter of the RNA structure, using isothermal titration calorimetry to measure heat evolution on binding SAM, and thus the affinity of the SAM ligand for the riboswitch. The underlying assumption is that the k-turn is integral to the structure of the riboswitch, and that SAM binding may be taken to indicate that the k-turn is correctly folded.

**Figure 3:**
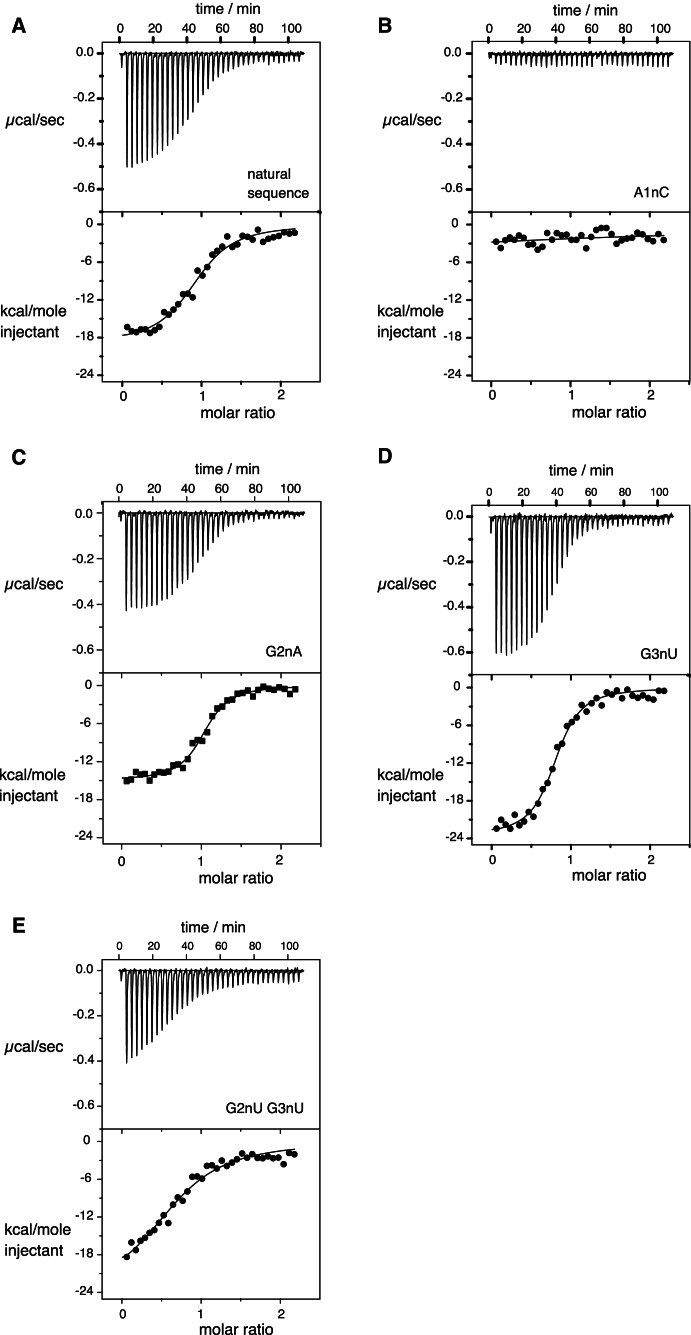
Isothermal Titration Analysis of SAM Binding to the Natural Sequence Riboswitch and Variants with k-Turn Substitutions A solution of SAM was titrated into a SAM-I riboswitch RNA solution, and the heat evolved was measured by ITC as the power required to maintain zero temperature difference with a reference cell. Integration over time gives the heat required to maintain thermal equilibrium between cells. In each case, the upper panel shows the raw data for sequential injections of 8 μl volumes (following an initial injection of 1 μl) of a 100 μM solution of SAM into a 1.4 ml 10 μM RNA solution in 50 mM HEPES (pH 7.5), 100 mM KCl, 10 mM MgCl_2_. This represents the differential of the total heat (i.e., enthalpy Δ*H*° under conditions of constant pressure) for each SAM concentration. The lower panels present the integrated heat data fitted (where possible) to a single-site binding model. The thermodynamic parameters calculated are summarized in Table 1. The ITC analysis was performed for the SAM-I riboswitch in which the k-turn sequence was modified as follows: (A) Unmodified k-turn. (B) The A1nC substitution. (C) The G2nA substitution. (D) The G3nU substitution. (E) The G2nU, G3nU double substitution. Titration data for further variants are presented in Figure S2.

Titration of SAM into a solution of the SAM-I riboswitch RNA is seen to be an exothermic process (Figure 3A). Fitting the data to a single-site binding model gave thermodynamic values for enthalpy and entropy (Table 1) from which we calculate Δ*G°* = −36 ± 1.2 kJ mol^-1^ and *K*_d_ = 0.54 ± 0.25 μM, similar to the values reported earlier by Batey and colleagues (Montange et al., 2010).

### Binding of SAM to the Riboswitch with Sequence Substitutions in the k-Turn

The calorimetry results confirm the binding of SAM to the unmodified riboswitch. We can now use this approach to ask if any sequence changes that prevent folding of the isolated k-turn can be accommodated within the complete riboswitch such that SAM binding is still possible.

Conversion of the k-turn into a simple 3 nucleotide bulge (A1nC, G2nU, G3nU variant) resulted in no detectable evolution of heat on addition SAM (Figure S2B). Evidently the complete removal of the k-turn has prevented the riboswitch from achieving the conformation in which it can bind its ligand. The single substitution at the 1b⋅1n position A1nC also resulted in no binding of SAM detectable by virtue of heat evolved (Figure 3B). Closely similar results were obtained for an A1nU substitution (Figure S2C). These single changes to the critical G⋅A pair are sufficient to destabilize the k-turn in the context of the riboswitch, and thus prevent the riboswitch from folding into its active conformation.

However, single-nucleotide substitutions at the second and third positions were less detrimental to ligand binding. The substitution G2nA prevented the folding of the isolated k-turn (see above). The same modification was introduced into the complete riboswitch, which was titrated with SAM (Figure 3C). In contrast to the riboswitch modified at the 1b⋅1n position, the G2nA variant clearly bound SAM in an exothermic process, with thermodynamic parameters presented in Table 1. The corresponding binding affinity *K*_d_ = 0.31 ± 0.06 μM is similar to that of the natural riboswitch. The conclusion that emerges is that the G2nA substitution that prevented the folding of the isolated k-turn is tolerated in the context of the riboswitch, indicating that the riboswitch structure has stabilized the variant k-turn that was not capable of folding in isolation. The riboswitch carrying a G3nU substitution in its k-turn also binds SAM exothermically (Figure 3D), with thermodynamic parameters similar to those of the natural riboswitch (Table 1). Thus the partial destabilization of the isolated k-turn does not impair the function of the riboswitch, and the folding of the k-turn is deduced to occur normally in the context of the complete RNA.

We have also analyzed the double substitution G2nU, G3nU in the context of the complete riboswitch, which was titrated with SAM (Figure 3E). This variant also clearly bound SAM in an exothermic process, with Δ*G°* = −33 ± 0.4 kJ mol^-1^ (Table 1). The corresponding binding affinity *K*_d_ = 1.95 ± 0.27 μM is about 4-fold weaker than that of the natural riboswitch. Nevertheless, this double substitution that prevented the folding of the isolated k-turn is tolerated in the context of the riboswitch.

### Crystal Structure of the SAM-I Riboswitch with a Modified k-Turn

Calorimetry shows that a SAM-I riboswitch containing a k-turn that lacks the G⋅A pair at the 2b⋅2n position can nevertheless bind SAM ligand, suggesting that the k-turn can fold in such a way to allow formation of a functional riboswitch. Does the k-turn fold into standard conformation, and if so, what is its local structure at the modified position? To address these questions, we have determined the crystal structure of the G2nA SAM-I riboswitch in complex with SAM. This provides a direct observation of the k-turn conformation in context of the riboswitch at close to atomic resolution.

The G2nA SAM-I riboswitch crystallized in conditions very similar to the unmodified riboswitch (see Experimental Procedures). The crystal was mounted on a cryoloop and diffracted to 2.6 Å. The space group P4_3_2_1_2 is retained and the unit cell parameters (Table S3) are virtually identical to those of the natural riboswitch (Montange et al., 2010), indicating that the crystals of natural and G2nA riboswitch are isomorphic. The initial phases were obtained by molecular replacement using the natural riboswitch as a model. To exclude the possibility of model bias that might significantly influence the derived structure, we calculated a composite omit map. This map (Figure S5) strongly supports our model and importantly all k-turn nucleotides are well defined in this map.

**Figure 4:**
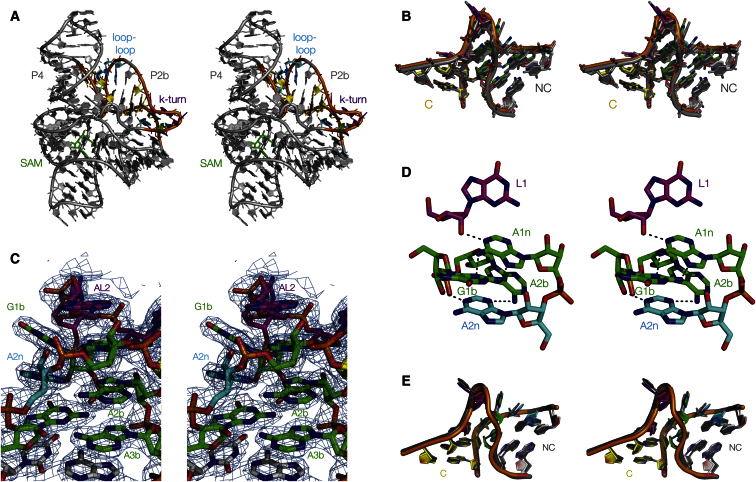
Parallel-Eye Stereo Views of the X-Ray Crystal Structure of the SAM-I Riboswitch Containing a G2nA Variant k-Turn (A) The structure of the complete G2nA SAM-I riboswitch bound to SAM. The SAM ligand is shown in green. The RNA is color coded equivalently to the cartoon shown in Figure 1C. (B) Superimposition of the k-turn structures (shown in cartoon form) of the natural (gray) and G2nA variant (color-coded) riboswitches. (C) 2**F**_obs_-**F**_calc_ electron density map of the k-turn of the G2nA variant riboswitch, contoured at 1 σ. The view shown is of the core region looking down onto the nonbulged strand. (D) Detail from the structure of the G2nA variant k-turn, showing the G⋅A and A⋅A base pairs with some important hydrogen bonds. The view is into the major groove of the NC helix. Further views are presented in Figure S3 and an omit map of the structure is shown in Figure S5. (E) Structure of the G2nU, G3nU k-turn within the SAM-I riboswitch. Superimposition of the k-turn structures (shown in cartoon form) of the natural (gray) and G2nU, G3nU variant (color coded) riboswitches. Further views are presented in Figures S4.

The overall fold and structure of the riboswitch (Figure 4A) is conserved in the variant, and no major deviations from the natural structure could be observed. The electron density for both the RNA and the SAM ligand is very well defined. Using the phosphorus atoms the natural and variant riboswitch structures were aligned with an rmsd of 0.74 Å.

Turning to the k-turn region of the structure specifically, the natural and variant structures could be superimposed well (using the phosphorus atoms of residues 17–22 and 30–38), with an rmsd of 0.53 Å (Figure 4B). Thus, the global fold of the variant k-turn is close to that of a standard k-turn structure, despite the presence of sequence changes that prevent folding of the same k-turn as an isolated duplex. Examination of the 2**F**_obs_-**F**_calc_ electron density map in the k-turn region (Figure 4C) shows that the positions of both the backbone and all the nucleobases are well defined by the electron density, and that all the interactions can be identified with high confidence. The hydrogen-bonding interactions in the core of the k-turn are shown in Figure 4D. The G2nA substitution creates a potential A⋅A base pair at the 2b⋅2n position, and we see that this pairing is stabilized by a single hydrogen bond from A2bN6 to A2nN3. Further stabilization should result from hydrogen bonds from G-1nO2′ to A2bN3, and from G1bO2′ to A2nN1. The A⋅A pair is stacked between the G⋅A pairs at the 1b⋅1n and 3n⋅3b positions, both of which are standard *trans* sugar-Hoogsteen G⋅A pairs stabilized by hydrogen bonding between GN2 to AN7 and AN6 to GN3. The near-universal interactions of the k-turn (Liu and Lilley, 2007) are preserved; L1O2′ to A1nN1 and L3O2′ to L1/L2 phosphate *pro*S O, as well as the bonding between A1nO2′ and AL2N6 which is frequently observed where L2 is adenine. The A-minor interactions between the G⋅A pairing region and the C helix are mediated by hydrogen bonding between A2bO2′ and G-1nN2, and the aforementioned G-1nO2′ to A2bN3, forming a zipper-like network (Figure S3). The former interaction is clearly sequence specific, and we have noted that these cross-helix interactions are variable between different k-turns (Liu and Lilley, 2007).

We have also solved the crystal structure for the SAM-I riboswitch in which the k-turn contains the double G2nU, G3nU substitution. As with the G2nA, this intrinsically unstable k-turn is stabilized in the context of the riboswitch. The crystals diffracted to 2.9 Å, but due to low completeness between 2.9 and 3.0 Å the data set is regarded as 3 Å resolution. The overall fold and structure of the riboswitch is again maintained (Figure S4A), with an rmsd from the natural riboswitch of <0.5 Å, the k-turn regions of the natural and variant riboswitches superimposed with an rmsd of 0.75 Å (Figure 4E), showing that the global structure of the k-turn was preserved in the riboswitch. However, while the complete backbone of the G2nU, G3nU k-turn structure is well defined by the electron density, some of the nucleobases in the core of the structure are relatively weakly defined at this resolution (Figure S4B), including the two uridine bases at the 2n and 3n positions, i.e., the two substituted nucleotides. This is consistent with the nucleobases being disordered due to increased flexibility but the experimental evidence is insufficient to confirm this interpretation. Nevertheless, it is clear that the G2nU, G3nU variant adopts the overall structure of a conventionally folded k-turn for which a model is provided in Figure S4C.

## Discussion

The SAM-I riboswitch contains a near-standard k-turn that plays an important role in the architecture of the functional riboswitch. The axial kink of the k-turn generates the correct trajectory of helix P2b that permits its terminal hairpin loop to make a loop-loop interaction with an acceptor in helix P4. Inserted into a simple helix, the k-turn folds normally on addition of Mg^2+^ ions in free solution, but folding is prevented by sequence substitutions that disrupt the G⋅A pairs at the 1b⋅1n and 2b⋅2n positions. In all respects, the behavior of the SAM-I riboswitch k-turn is closely similar to that of a standard k-turn such as *H. marismortui* Kt-7.

We observed that the substitutions G2nA (creating a potential A⋅A pair at the 2b⋅2n position) and G2nU, G3nU (disrupting both the 2b⋅2n and 3b⋅3n pairs) prevented significant folding into the kinked structure in the isolated helical RNA. Yet, those same substitutions within the SAM-I riboswitch did not prevent SAM binding. This suggested that in the context of the more complex RNA structure, the k-turn could fold despite the disruption of the 2b⋅2n and 3b⋅3n pairs. This was confirmed more directly by solving the X-ray crystal structures of the two variant riboswitches, revealing that the modified k-turn adopts a standard k-turn fold in both cases. Thus sequence substitutions that prevent metal ion-induced folding of the isolated RNA do not prevent folding within the larger structure of the riboswitch. Evidently, the tertiary interactions in the riboswitch “force” the impaired k-turns to fold. There is likely to be some overall stabilization resulting from binding the SAM ligand to the riboswitch. But since this occurs at a site that is remote from the k-turn or loop-loop interaction the stabilization of the k-turn must be mediated through the tertiary structure of the RNA. The overall free energy of riboswitch folding is sufficient to stabilize some compromised k-turn structures. By contrast, the A1nC- or A1nU-substituted riboswitches did not bind SAM, indicating that these variants could not be induced to fold by the overall RNA structure. Disruption of the 1b⋅1n is clearly more destabilizing, such that the free energy of riboswitch folding is insufficient to overcome the destabilization of the k-turn in this case. Further support for the stabilization of the k-turn in the context of the riboswitch is provided by NAIM experiments of Hennelly and Sanbonmatsu (2011), suggesting that an inosine substitution at the 2n position of the k-turn does not prevent population of the folded fraction. Another example of the stabilization of a kinked (but not k-turn) structure by tertiary structure has been provided by Strobel and colleagues in a group I intron structure (Antonioli et al., 2010).

The crystal structures of the G2nA and G2nU, G3nU riboswitches confirm that they are folded into a structure that superimposes well with the unmodified k-turn, and with well-studied k-turns like *H. marismortui* Kt-7. Unfortunately the resolution of the G2nU, G3nU riboswitch is insufficient to be confident about the interactions stabilizing the core of that structure. However, the positions of all the nucleobases of the G2nA riboswitch are very well defined in that structure, and so the hydrogen bonding can be identified with good confidence. All the standard, well-conserved hydrogen bonds are present, and the adenine nucleobases at the 1n and 2b positions are placed correctly so that A-minor interactions between the minor groove edges of the C and NC helices are formed. The variant sequence forms an A⋅A pair at the 2b⋅2n position, held in place by a number of hydrogen bonds, and this study provides the first crystallographic structure of a standard k-turn with an A⋅A base pair at this position. Thus the A⋅A pair is evidently not sufficient to permit the stable formation of this k-turn in isolation, yet the k-turn can fold when stabilized by the tertiary interactions. Interestingly, an A⋅A pair occurs naturally at the 2b⋅2n position of the sequence of *T. solenopsae* Kt-23 in the small ribosomal subunit, and can be induced to fold by the binding of L7Ae protein. At present there is no structure available for this k-turn, but a crystallographic study of this RNA is underway in this laboratory, and it will be interesting to see if the structure formed in Kt-23 is the same as that observed here. In principle, an A⋅U pair could form at the 2b⋅2n position of the G2nU, G3nU variant, but we cannot be sure that it does so on the basis of the present structure for that riboswitch. In some natural k-turn sequences, the usual G at the 2n position is replaced by U, exemplified by Kt-23 of the *T. thermophilus* 16S rRNA. In that case, U2n forms a reverse Hoogsteen A⋅U pair with the A2b (Wimberly et al., 2000), and the k-turn can fold in an isolated helix by addition of Mg^2+^ ions (Schroeder and Lilley, 2009) unaided by tertiary interactions.

In the light of these results, we can now list three ways in which the kinked conformation of the k-turn may be stabilized:

1. In isolation, most k-turns become stabilized by the addition of metal ions, at ∼100 μM for divalent and ∼30 mM for monovalent ions (Goody et al., 2004; Liu and Lilley, 2007).
2. The binding L7Ae and related proteins has been shown to induce the formation of k-turns (Turner and Lilley, 2008; Turner et al., 2005), even in the absence of metal ions.
3. We now show that the tertiary structure of larger RNA structures can stabilize the folded structure of k-turns, overcoming substitutions that prevent folding in isolated RNA duplexes.

Evidently k-turn structures can exert a significant influence on the long-range architecture of larger RNA assemblies; the SAM-I riboswitch illustrates this very well on a relatively small scale. It is therefore conceivable that k-turns could play an important role in the formation of larger RNA structures such as a ribosomal subunit. It is likely that the stabilization of k-turns by both protein binding and tertiary interactions could be involved during the biogenesis of such structures.

## Experimental Procedures

### RNA Preparation

RNA oligonucleotides were synthesized using *t*-BDMS phosphoramidite chemistry (Beaucage and Caruthers, 1981), as described in Wilson et al. (2001). SAM-I RNA was transcribed from a PCR-amplified template and purified by gel electrophoresis under denaturing conditions.

### Fluorescence Spectroscopy

Fluorescence spectra were recorded in 90 mM Tris-borate (pH 8.3) at 4°C using an SLM-Aminco 8100 fluorimeter. Values of FRET efficiency (*E*_FRET_) were measured using the acceptor normalization method (Clegg, 1992) implemented in MATLAB. *E*_FRET_ as a function of metal ion concentration was analyzed on the basis of a model in which the fraction of folded molecules corresponds to a simple two-state model for ion-induced folding, i.e.,

[1] $$E_{\text{FRET}}=E_{\text{0}}+\frac{\Delta E_{\text{FRET}}.K_{a}{\left[\text{M}\right]}^{n}}{1+K_{\text{a}}{\left[\text{M}\right]}^{n}},$$

where *E*_0_ is the FRET efficiency of the RNA in the absence of added metal ions, *ΔE*_FRET_ is the increase in FRET efficiency at saturating metal ion concentration, [M] is the prevailing Mg^2+^ ion concentration, *K*_a_ is the apparent association constant for metal ion binding and *n* is a Hill coefficient.

The sequences used in the FRET analyses were

bulged strand: Fluorescein-CCAGUCAGUCCCGACGAAACCUGUCAGG
nonbulged strand: Cy3-CCUGACAGGUGGAGGGACUGACUGG

Nucleotide substitutions were introduced as required.

### Isothermal Titration Calorimetry

Microcalorimetric measurements of SAM binding to the SAM-I riboswitch and variants were performed by isothermal titration calorimetry (ITC) as described by Montange et al. (2010). The sequence of the SAM-I riboswitch was GGCUUAUCAAGAGAGGUGGAGGGACUGGCCCGACGAAACCCGGCAACCAGAAAUGGUGCCAAUUCCUGCAGCGGAAACGUUGAAAGAUGAGCCG together with substitutions noted in the text. Calorimetric data were fitted to a single-site binding model, where possible, using MicroCal ORIGIN software. Individual heat changes Δ*Q* at constant pressure are given by

[2] $$\Delta Q=\text{v}.\Delta H.\left[\text{RNA}\right].\left\{(\frac{K_{\text{a}}.{\left[\text{SAM}\right]}_{\text{i}}^{\text{n}}}{1+K_{\text{a}}.{\left[\text{SAM}\right]}_{\text{i}}^{\text{n}}})-\left(\frac{K_{\text{a}}.{\left[\text{SAM}\right]}_{\text{i}-1}^{\text{n}}}{1+K_{\text{a}}.{\left[\text{SAM}\right]}_{\text{i}-1}^{\text{n}}}\right)\right\},$$

where Δ*H* is the change in enthalpy, v is the reaction volume, *K*_a_ is the association constant for SAM binding, and [SAM]_i_ is the SAM concentration at the *i* th injection.

### X-Ray Crystallography

The SAM-I riboswitch variants were crystallized using the hanging drop method. Diffraction data were collected on ID14-4 (G2nU, G3nU) and BM-14 (G2nA) at the European Synchrotron Radiation Facility in Grenoble, France. The structure of G2nU, G3nU was solved by performing a rigid body refinement using REFMAC5 (Vagin et al., 2004) with the RNA plus SAM-ligand structure PDB entry 3gx5 (Montange et al., 2010) as a preliminary model. The G2nA structure was solved using MolRep with PDB entry 3gx5 as the starting model.

Fuller experimental details are presented in the Supplemental Experimental Procedures.

## Figures and Tables

**Table 1:** Thermodynamic Parameters Calculated for the SAM-I Riboswitch with and without Sequence Variations in the k-Turn

k-Turn	*n*	Δ*H*° / kJ mol^-1^	Δ*S*°/ J mol^-1^ K^-1^	Δ*G*°/ kJ mol^-1^	*K*_d_/ μM
Natural	0.85 ± 0.10	−74 ± 5	−120 ± 22	−36 ± 1.2	0.54 ± 0.25
A1nC	ND	ND	ND	ND	no binding
G2nA	1.0 ± 0.1	−59 ± 5	−71 ± 13	−38 ± 0.5	0.31 ± 0.06
G3nU	0.7 ± 0.1	−98 ± 8	−200 ± 23	−38 ± 0.3	0.34 ± 0.04
G2nU, G3nU	0.7 ± 0.1	−100 ± 18	−220 ± 64	−33 ± 0.4	1.95 ± 0.27

ND, not determined because no heat evolved.

## Accession Numbers

Coordinates have been deposited in the Protein Data Bank with accession codes 2ygh and 2ydh.

## Supplemental Information

Document S1. Five Figures, Four Tables, and Supplemental Experimental Procedures
