# Supplementary material for: RNA Tertiary Interactions in a Riboswitch Stabilize the Structure of a Kink Turn
Source: Structure. 2011 Sep 7;19(9):1233–40. doi: 10.1016/j.str.2011.07.003 (PMC3651934; doi:10.1016/j.str.2011.07.003)
Supplement: Document S1. Five Figures, Four Tables, and Supplemental Experimental Procedures [file mmc1.pdf]

Structure *19*

Supplemental Information

RNA Tertiary Interactions in a Riboswitch

Stabilize the Structure of a Kink Turn

Kersten T. Schroeder, Peter Daldrop, and David M. J. Lilley

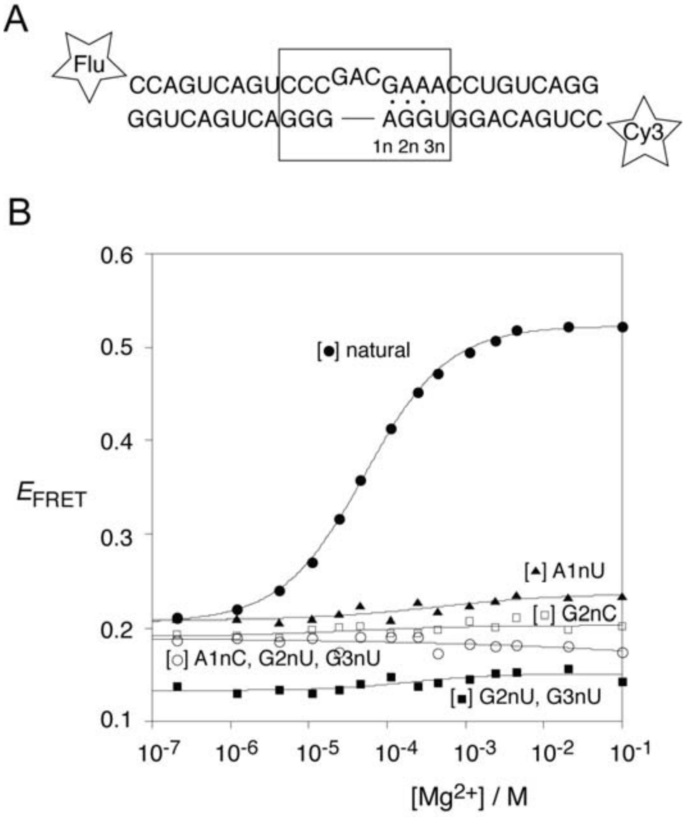

Figure S1, related to Figure 2. Folding of the SAM-I riboswitch k-turn and further sequence variants as isolated RNA duplexes.

The k-turn sequence and variants weres located centrally in a 25 bp duplex, terminally labeled with fluorescein (Flu) and Cy3 (Cy3) fluorophores. FRET efficiency (E_{FRET}) was measured in the steady-state as a function of Mg^{2+} ion concentration.

A. The sequence of the RNA duplex containing the natural sequence k-turn. Other substitutions were included as indicated.

B. Plot of E_{FRET} as a function of Mg^{2+} ion concentration. The data have been fitted to a model for a two-state transition (equation (1)) (lines). Data : Filled circles, natural sequence k-turn; filled triangles, A1nU substitution; open squares G2nC substitution; filled squares, G2nU, G3nU double substitution; open circles, A1nC, G2nU, G3nU triple substitution. Note that the A1nC, G2nU, G3nU substitution converts the k-turn into a simple three-nucleotide bulge.

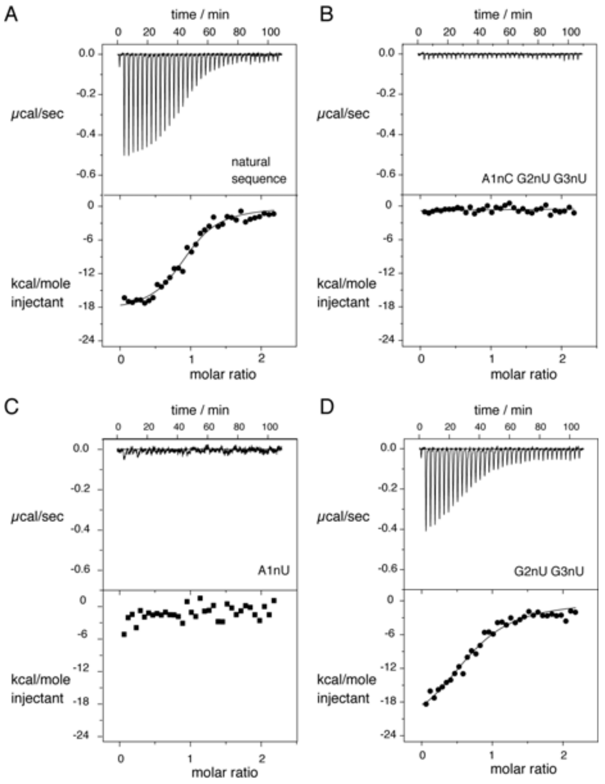

Figure S2, related to Figure 3. Isothermal titration analysis of SAM binding to the natural sequence riboswitch and further variants with k-turn substitutions.

A solution of SAM was titrated into a SAM-I riboswitch RNA solution, and the heat evolved was measured by ITC as the power required to maintain zero temperature difference with a reference cell. Integration over time gives the heat required to maintain thermal equilibrium between cells. In each case the upper panel shows the raw data for sequential injections of 8 μL volumes (following an initial injection of 1 μL) of a 100 μM solution of SAM into a 1.4 mL 10 μM RNA solution in 50 mM HEPES (pH 7.5), 100 mM KCl, 10 mM $MgCl_2$. This represents the differential of the total heat (ie enthalpy $\Delta H°$ under conditions of constant pressure) for each SAM concentration. The lower panels present the integrated heat data fitted (where possible) to a single-site binding model. The thermodynamic parameters calculated are summarized in Table S2. The ITC analysis was performed for the SAM-I riboswitch in which the k-turn sequence was modified as follows:

A. Unmodified k-turn.

B. The A1nC, G2nU, G3nU triple substitution. These changes convert the k-turn into a simple three-nucleotide bulge.

C. The A1nU substitution.

D. The G2nU, G3nU double substitution.

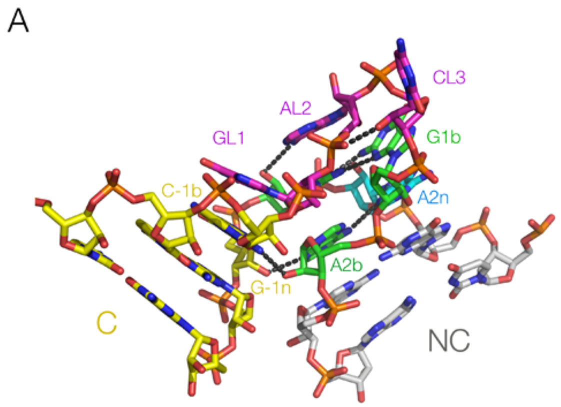

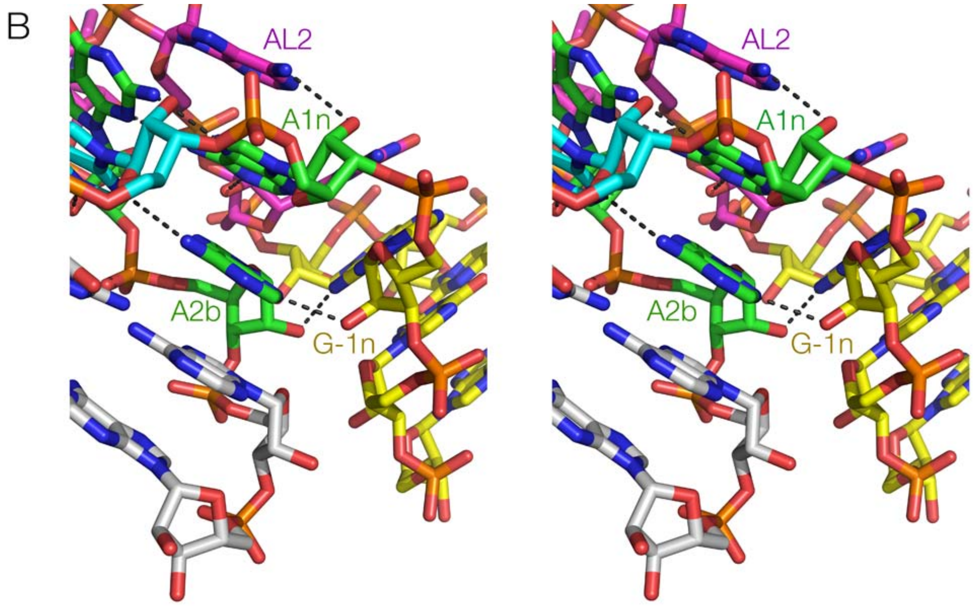

Figure S3, related to Figure 4. Two further parallel-eye stereo views of the X-ray crystal structure of the SAM-I riboswitch k-turn containing a G2nA variant k-turn.

A. View of the k-turn structure observed from the side of the bulged strand.

B. Close-up view of the A-minor interactions between the C and NC helices observed from the side of the non-bulged strand.

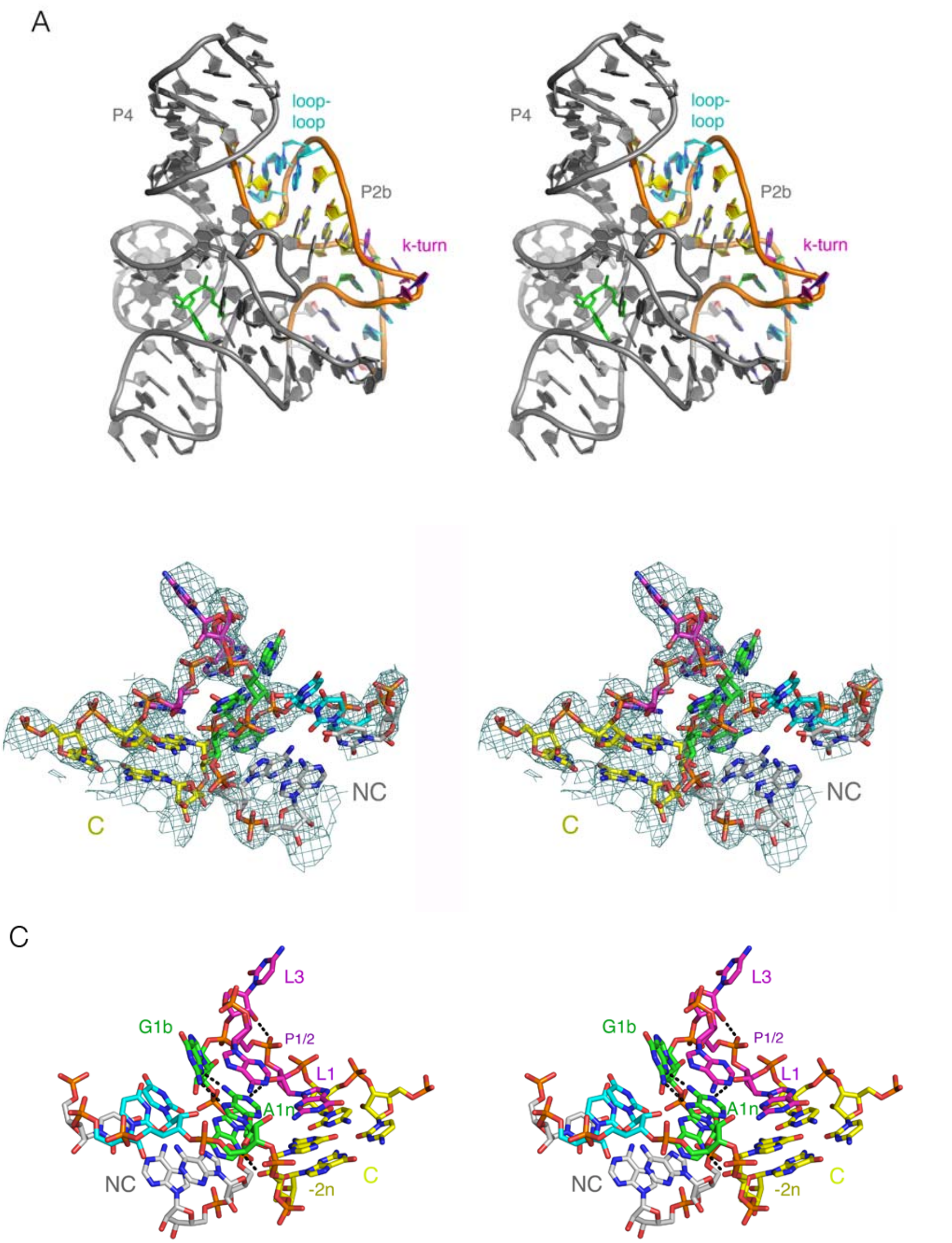

Figure S4, related to Figure 4E. Parallel-eye stereo views of the X-ray crystal structure of the SAM-I riboswitch containing a G2nU, G3nU variant k-turn.

A. The structure of the complete G2nU, G3nU SAM-I riboswitch bound to SAM. The SAM ligand is shown in green. The RNA is color coded to match the cartoon shown in Figure 1C.

B. 2$\mathbf{F}_{obs}$-$\mathbf{F}_{calc}$ electron density map of the k-turn of the G2nU, G3nU variant riboswitch, contoured at 1 σ. The view shown is approximately from the side of the bulged strand.

C. Model of the k-turn of the G2nU, G3nU variant k-turn, with important hydrogen bonds shown. The view is approximately from the side of the non-bulged strand, ie opposite to that in part (B).

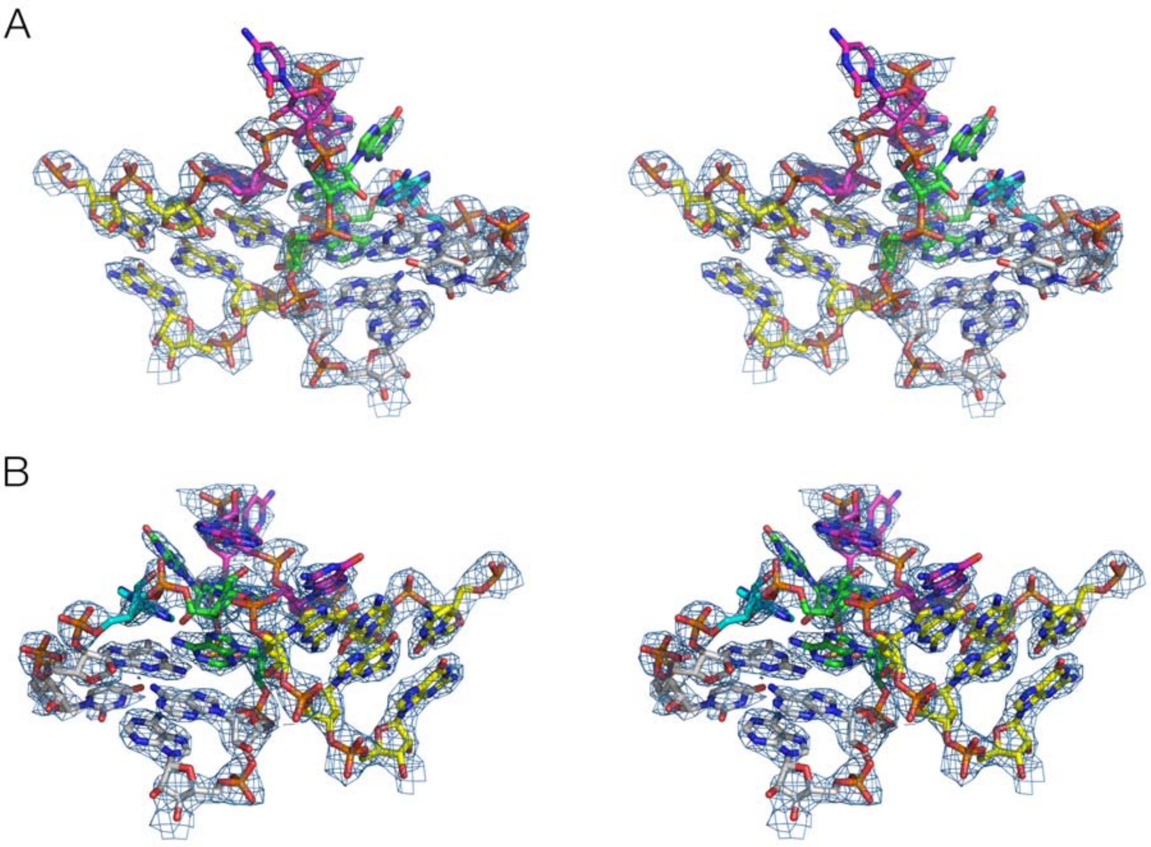

Figure S5, related to Figure 4. Parallel-eye stereo views of an omit map calculated for the the SAM-I riboswitch containing a G2nA variant k-turn.

Omit map of the k-turn region of the G2nA SAM-I riboswitch at 1.2 σ calculated using PHENIX. Two stereo views are shown, from the side of the bulge-containing strand (**A**), and that of the non-bulged strand (**B**). The backbone of all the nucleotides is well defined by the omit map, and the nucleobase region is also very well defined for nearly all nucleotides. This strongly supports our model without the potential model bias associated with 2$\mathbf{F}_{obs}$-$\mathbf{F}_{calc}$ maps.

\

Table S1.

k turn	n	$[Mg^{2+}]_{1/2}$ / μM	ΔE_{FRET}
Natural	0.8 ± 0.1	50 ± 8	0.32 ± 0.03
A1nC, G2nU, G3nU	-	-	NF
G2nU, G3nU	-	-	NF
G3nU	0.9 ± 0.1	110 ± 12	0.19 ± 0.02
A1nC	-	-	NF
A1nU	-	-	NF
A1nG	-	-	NF
G2nC	-	-	NF
G2nA	-		NF
G2nU	-	-	NF
G1bA	-	-	NF

Table S1. Summary of the folding properties of the SAM-I k-turn and sequence variants analyzed as duplex species by FRET as a function of Mg2+ concentration (see Figure 2). The isotherms were fitted to a two-state model of ion-induced binding. The tabulated parameters were calculated from the fits : n, the Hill coefficient; $[Mg^{2+}]_{1/2}$, the Mg^{2+} concentration at the mid-point of the transition; ΔE_{FRET} the change in FRET efficiency over the folding transition. NF denotes no detectable folding. Although we have fitted the G2nA to a transition, the amplitude is very small and requires a ten-fold higher Mg^{2+} concentration. This can therefore be regarded as essentially not folded.

Table S2.

k-turn	n	$\Delta H°$ / kJ mol^{-1}	$\Delta S°$ / J mol^{-1} K^{-1}	$\Delta G°$ / kJ mol^{-1}	K_d / μM
natural	0.8 ± 0.1	-73 ± 5	-120 ± 22	-36 ± 1.2	0.54 ± 0.25
A1nC, G2nU, G3nU	ND	ND	ND	ND	no binding
A1nC	ND	ND	ND	ND	no binding
A1nU	ND	ND	ND	ND	no binding
G2nA	1.0 ± 0.1	-59 ± 5	-71 ± 14	-38 ± 0.5	0.31 ± 0.06
G2nC	ND	ND	ND	ND	no binding
G2nU, G3nU	0.7 ± 0.1	-100 ± 18	-220 ± 640	-33 ± 0.4	1.95 ± 0.27
G3nU	0.7 ± 0.1	-98 ± 8	-200 ± 23	-38 ± 0.3	0.34 ± 0.04

ND = not determined because no heat evolved.

Table S2. Thermodynamic parameters calculated for SAM binding to the SAM-I riboswitch and sequence variants in the k-turn.

Table S3.

	details of data collection	
sequence	G2nA	G2nU, G3nU
PDB code	2ygh	2ydh
space group	$P4_32_12$	$P4_32_12$
unit cell dimensions / Å	a = 58.46<br>b = 58.46<br>c = 155.26<br>α = β = γ = 90.00°	a = 61.89<br>b = 61.89<br>c = 157.63<br>α = β = γ = 90.00°
resolution range / Å	51.8-2.60<br>(2.74-2.6)	50-2.9<br>(3.00-2.90)
observations	82286	41985
unique observations	8929	6875
completeness (%)	100.0 (100.0)	93.1 (64.5)
<I/σ(I)>	22.6 (6.0)	40.6 (2.44)
R_{merge} (%)	7.0 (38.3)	5.1 (42.3)
Multiplicity	9.2 (9.5)	6.1(5.6)
Number of molecules in the asymmetric unit / Mathews coefficient	1 / 2.16	1 / 2.31
	**refinement statistics**	
resolution range / Å	51.8-2.6	50-2.9
R-factor % (R_{work}/R_{free})	20.0/25.9	26.9/32.2
number of atoms[a]	2033/27/6/19	2024/27/5/2
mean *B*-factor[b] (Å^2)	28.8/21.3/25.0/17.0	96.9/77.3/161.7/30
RMS bond length deviation / Å	0.01	0.01
RMS bond angle deviation / °	1.892	1.914

[a] number of atoms for RNA, ligand, ions and water molecules.

[b] mean *B*-factors for RNA, ligand, ions and water molecules, respectively.

Table S3. Crystallographic parameters for the SAM-I riboswitches with G2A and G2nU, G3nU substitution in the k-turn.

Table S4.

	unmodified	G2nA	G2nU, G3nU
PDB code	3gx5	2ygh	2ydh
All-atom contacts (clash score/percentile)	25.4 / 44	6.78 / 99	14.26 / 96
Probably wrong sugar puckers	7	1	4
Bad backbone conformations	15	8	22
Nucleotides with bad bonds	0.00%	3.19%	2.13%
Nucleotides with bad angles	10.64%	35.11%	35.11%

Table S4: Geometric analysis of crystal structures

The geometric analysis of the structures calculated using MolProbity are comparable with that of the previously reported natural structure. The clash score of the stuctures presented is better than that of the natural sequence which is also reflected in the 97th and 99th percentile for these structures compared with the 44th percentile for the natural structure. Similarly both structures presented here perform better in terms of the sugar puckers. For the backbone conformations the G2nA structure performs better than the natural structure while the G2nU, G3nU structure performs less well. Both in terms of bond length and angle deviations the natural structure reported by Montange and coworkers (2010) yields better statistics than the structures reported in this work. In conclusion the mutant structures perform overall comparable with the previously reported natural structure.

SUPPLEMENTAL EXPERIMENTAL PROCEDURES

RNA preparation

RNA oligonucleotides were synthesized using *t*-BDMS phosphoramidite chemistry (Beaucage and Caruthers, 1981), as described in Wilson et al. (Wilson et al., 2001). Fluorescein and Cy3-conjugated oligonucleotides were attached at 5'-termini as phosphoramidites during synthesis as required. Oligoribonucleotides were deprotected in 25% ethanol/ammonia solution at 65°C for 1 h, and evaporated to dryness. They were redissolved in 300 µL 1 M tetrabutylammonium fluoride (Aldrich) in tetrahydrofuran to remove *t*-BDMS protecting groups, and agitated at 20°C for 16 h prior to desalting by G25 Sephadex (NAP columns, Pharmacia) and ethanol precipitation. All oligonucleotides

were purified by gel electrophoresis in polyacrylamide, and recovered from gel fragments by electroelution or diffusion in buffer followed by ethanol precipitation. Fluorescently-labeled RNA was subjected to further purification by reversed-phase HPLC on a C18 column (ACE10, Advanced Chromatography Technologies) using an acetonitrile gradient with an aqueous phase of 100 mM triethylammonium acetate pH 7.0. Duplex species were annealed in 50 mM Tris-HCl (pH 7.5), 25 mM NaCl by slow cooling from 90 to 4°C, and purified by electrophoresis.

SAM-I RNA (having the sequence from the PDB file 3gx5) was transcribed from a PCR-amplified template. The PCR was carried out using the primers (all sequences written 5' to 3'):
GCGCGCGAATTCTAATACGACTCACTATAG as 5' primer
CGGCTCATCTTTCAACGTTTCCGCT as 3'primer
The PCR reaction contained: 20 mM Tris-HCl (pH 8.8), 10 mM $(NH_4)_2SO_4$, 10 mM KCl, 2 mM $MgSO_4$, 0.1 % Triton X-100, 0.2 mM each dNTPs, 1 μM each primer, 600 ng/ml SAM-I plasmid with the appropriate mutations (natural SAM-I sequence supplied by R. Batey, with substitutions as required generated by site-directed mutagenesis) and 1 U/mL Taq polymerase (New England Biolabs). Transcription reactions contained 40 mM Tris-HCl (pH 8.0), 4 mM each NTP (Sigma), 32 mM $MgCl_2$, 10 mM DTT, 3.2 mM spermidine, 0.01% Triton X-100, 0.25 mg/ml T7-RNA polymerase, 8.0% (v/v) DNA template from the PCR reaction and 0.1 U/mL pyrophosphatase (Sigma). Reactions were incubated at 37°C and precipitated with 3 reaction volumes of ethanol. Transcribed RNA was purified on 12% polyacrylamide (19:1) gels in presence of 90 mM Tris.borate (pH 8.3), 10 mM EDTA, 7 M urea.

Fluorescence spectroscopy

Absorption spectra were measured in 90 mM Tris-borate (pH 8.3) in 120 μL volumes using a NanoDrop 2000C spectrophotometer (Thermo Scientific). Spectra were deconvoluted using a corresponding RNA species labeled only with Cy3, and fluorophore absorption ratios calculated using a MATLAB program. Fluorescence spectra were recorded in 90 mM Tris-borate (pH 8.3) at 4°C using an SLM-Aminco 8100 fluorimeter. Spectra were corrected for lamp fluctuations and instrumental variations, and polarization artifacts were avoided by setting excitation and emission polarizers crossed at 54.7°. Values of FRET efficiency (E_{FRET}) were measured using the acceptor normalization method (Clegg, 1992) implemented in MATLAB. E_{FRET} as a function of metal ion concentration was analyzed on the basis of a model in which the fraction of folded

molecules corresponds to a simple two-state model for ion-induced folding, ie

$$E_{\mathrm{FRET}} = E_0 + \Delta E_{\mathrm{FRET}} \,.\, K_a\,[\mathrm{M}]^n/(1 + K_{\mathrm{a}}\,[\mathrm{M}]^n) \quad \textbf{[1]}$$

where E_0 is the FRET efficiency of the RNA in the absence of added metal ions, ΔE_{FRET} is the increase in FRET efficiency at saturating metal ion concentration, [M] is the prevailing Mg^{2+} ion concentration, K_{a} is the apparent association constant for metal ion binding and n is a Hill coefficient. Data were fitted to this equation by nonlinear regression. The metal ion concentration at which the transition is half complete is given by $[\mathrm{M}]_{1/2} = (1/K_A)^{1/n}$.

The sequences used in the FRET analyses were :
bulged strand: Fluorescein-CCAGUCAGUCCCGACGAAACCUGUCAGG
non-bulged strand: Cy3-CCUGACAGGUGGAGGGACUGACUGG
Nucleotide substitutions were introduced as required.

Isothermal titration calorimetry

Microcalorimetric measurements of SAM binding to the SAM-I riboswitch and variants were performed by isothermal titration calorimetry (ITC) using a MicroCal VP-ITC (GE Healthcare) at 30°C as described by Montange et al (Montange et al., 2010). The sequence of the SAM-I riboswitch was
GGCUUAUCAAGAGAGGUGGAGGGACUGGCCCGACGAAACCCGGCAACCAG
AAAUGGUGCCAAUUCCUGCAGCGGAAACGUUGAAAGAUGAGCCG
together with substitutions noted in the text. This sequence has two modifications used to aid crystallization, but we term this the natural sequence in this paper. A solution of 100 µM SAM (Sigma) were injected into the cell containing 1.4 mL of 10 µM SAM-I riboswitch RNA that had been dialyzed against 50 mM HEPES (pH 7.5), 100 mM KCl, 10 mM $MgCl_2$. Following a preliminary 1 µL injection, 35 repetitions of 8 µL aliquots of SAM were injected. Calorimetric data were fitted to a single-site binding model, where possible, using MicroCal ORIGIN software. Individual heat changes ΔQ at constant pressure are given by :

$$\Delta Q = \mathrm{v}.\ \Delta H.[\mathrm{RNA}].\{(K_{\mathrm{a}}.\ [\mathrm{SAM}]_{\mathrm{i}}^{\mathrm{n}} / 1{+}K_{\mathrm{a}}.\ [\mathrm{SAM}]_{\mathrm{i}}^{\mathrm{n}}) - (\ K_{\mathrm{a}}.\ [\mathrm{SAM}]^{\mathrm{n}}_{\mathrm{i-1}} / 1{+}K_{\mathrm{a}}.\ [\mathrm{SAM}]^{\mathrm{n}}_{\mathrm{i-1}})\} \quad \textbf{[2]}$$

where ΔH is the change in enthalpy, v is the reaction volume, K_{a} is the association constant for SAM binding, and $[\mathrm{SAM}]_{\mathrm{i}}$ is the SAM concentration at the i th injection

Each titration was performed three times and $\Delta H°$ and $\Delta S°$ values were averaged. Free

energy ($\Delta G°$) and the dissociation constant for SAM binding (K_d) were calculated from :

$$\Delta H° - T\Delta S° = \Delta G° = -RT\ln K_d \quad \textbf{[3]}$$

where R is the gas constant and T is the temperature (K).

X-ray crystallography

The SAM-I riboswitch variants were crystallized using the hanging drop method. Crystal plates were set up by mixing 1 μL of mother liquor with 1 μL of 400 μM RNA plus 1 mM SAM (Sigma Aldrich) in 40 mM Na-cacodylate (pH 7.0). Drops were seeded using the micro seeding technique with a seed stock obtained from crystal plates containing the unmodified RNA. The mother liquor of the drop that yielded the crystal of the G2nU, G3nU variant used for data collection contained 40 mM Na-cacodylate (pH 7.0), 12 mM spermine-HCl, 12% (v/v) MPD and 80 mM KCl. The crystal of the G2nA variant used for data collection was grown in 40 mM Na cacodylate (pH 7.0), 10 mM $MgCl_2$, 80 mM KCl, 16 mM spermidine HCl, 14 % (v/v) MPD. Crystals were grown at 20 °C. For data collection the crystal of the G2nU, G3nU variant was cryo-protected using the relevant well solution with 15% (v/v) PEG, while that of the G2nA variant was cryoprotected with the relevant well solution containing 30% MPD. Cryoprotectant was applied for approximately one minute before freezing the crystal in liquid nitrogen. Diffraction data were collected on ID14-4 (G2nU, G3nU) and BM-14 (G2nA) at the European Synchrotron Radiation Facility in Grenoble, France. Data were indexed, integrated and scaled using HKL2000 (Otwinowski et al., 2003) for G2nU, G3nU and MOSFLM/Scala for the G2nA structure from the CCP4 suite of programs (CCP4, 1994). Both structures were solved by performing a molecular replacement using with the RNA plus SAM-ligand structure PDB entry 3gx5 (Montange et al., 2010) as a preliminary model. The structures were refined using REFMAC5 from the CCP4 suite of programs, and the model was built using COOT (Emsley and Cowtan, 2004). The composite omit map was calculated using Phenix (Adams et al, 2010)

Supplemental References

P. D. Adams, P. V. Afonine, et al (2010). PHENIX: a comprehensive Python-based system for macromolecular structure solution. Acta Cryst. *D66*, 213-221.

Beaucage, S. L., and Caruthers, M. H. (1981). Deoxynucleoside phosphoramidites - a new class of key intermediates for deoxypolynucleotide synthesis. Tetrahedron Letters *22*, 1859-1862.

CCP4 (1994). The CCP4 suite: programs for protein crystallography. Acta Crystallogr D Biol Crystallogr *50*, 760-763.

Clegg, R. M. (1992). Fluorescence resonance energy transfer and nucleic acids. Meth Enzymol *211*, 353-388.

Emsley, P., and Cowtan, K. (2004). Coot: model-building tools for molecular graphics. Acta Crystallogr D Biol Crystallogr *60*, 2126-2132.

Montange, R. K., Mondragon, E., van Tyne, D., Garst, A. D., Ceres, P., and Batey, R. T. (2010). Discrimination between closely related cellular metabolites by the SAM-I riboswitch. J Molec Biol *396*, 761-772.

Otwinowski, Z., Borek, D., Majewski, W., and Minor, W. (2003). Multiparametric scaling of diffraction intensities. Acta Crystallogr A *59*, 228-234.

Wilson, T. J., Zhao, Z.-Y., Maxwell, K., Kontogiannis, L., and Lilley, D. M. J. (2001). Importance of specific nucleotides in the folding of the natural form of the hairpin ribozyme. Biochemistry *40*, 2291-2302.
